# Safety and reactogenicity of primary vaccination with the 10-valent pneumococcal non-typeable *Haemophilus influenzae* protein D conjugate vaccine in Vietnamese infants: a randomised, controlled trial

**DOI:** 10.1186/1471-2334-13-95

**Published:** 2013-02-21

**Authors:** Tran Ngoc Huu, Nguyen Trong Toan, Ha Manh Tuan, Ho Lu Viet, Pham Le Thanh Binh, Ta-Wen Yu, Fakrudeen Shafi, Ahsan Habib, Dorota Borys

**Affiliations:** 1 Pasteur Institute Ho Chi Minh City, 167 Pasteur Street District, 3 Ho Chi Minh City, Vietnam; 2 Children’s Hospital 2, Ho Chi Minh City, Vietnam; 3 GlaxoSmithKline Vaccines, 20 Avenue Fleming, 1300, Wavre, Belgium; 4 GlaxoSmithKline Pharmaceuticals, 5 Embassy Links, SRT Road, 560 052, Bangalore, India

**Keywords:** Pneumococcal conjugate vaccine, Infant immunisation, Safety, Reactogenicity, Vietnam, Co-administration

## Abstract

**Background:**

Pneumococcal infections are major causes of child mortality and morbidity worldwide and antibiotic resistance of *Streptococcus pneumoniae* is a major concern, especially in Asian countries. The present study was designed to evaluate the reactogenicity and safety of the 10-valent pneumococcal non-typeable *Haemophilus influenzae* protein D conjugate vaccine (PHiD-CV) when co-administered with the licensed diphtheria, tetanus, acellular pertussis, hepatitis B virus, inactivated poliovirus and *H. influenzae* type b vaccine (DTPa-HBV-IPV/Hib) in a 3-dose primary vaccination course in Vietnamese infants.

**Methods:**

This phase III, open, randomised study was conducted in one centre in Ho Chi Minh City between February and July 2011. Healthy infants (N=300) were randomised (2:1) to receive either PHiD-CV co-administered with DTPa-HBV-IPV/Hib (PHiD-CV group) or DTPa-HBV-IPV/Hib alone (Control group) at 2, 3, and 4 months of age.

**Results:**

Within 31 days post-vaccination, 8.2% of overall doses in the PHiD-CV group and 3.0% of overall doses in the Control group were followed by at least one solicited and/or unsolicited, local and/or general adverse event of grade 3 intensity. Pain at injection site was the most common grade 3 solicited symptom, which was reported following 6.5% and 1.0% of overall doses in the PHiD-CV and Control groups, respectively. Within 4 days post-vaccination, the most common solicited local and general symptoms reported with any intensity were pain (48.9% and 31.0% of doses in the PHiD-CV and Control groups) and irritability (58.0% and 40.4% of doses in the PHiD-CV and Control groups). Within 31 days post-vaccination, the incidence of unsolicited symptoms was comparable in both groups (following 12.3% and 14.8% of doses in the PHiD-CV and Control groups, respectively). Throughout the study, 13 serious adverse events (SAEs) were reported in 9 infants in the PHiD-CV group and 11 SAEs in 6 infants in the Control group. None of them were fatal or considered causally related to vaccination.

**Conclusions:**

PHiD-CV had a clinically acceptable safety profile when co-administered with DTPa-HBV-IPV/Hib in Vietnamese infants. The reactogenicity of PHiD-CV was comparable to that observed in other South-East Asian populations.

**Trial registration:**

ClinicalTrials.gov: http://NCT01153841

## Background

Pneumococcal infections are a major cause of child mortality and morbidity worldwide, especially in developing countries [1]. In Vietnam, the incidence of invasive pneumococcal disease was estimated to be at least 48.7 cases per 100,000 children younger than 5 years of age in a previous study conducted in Khanh Hoa Province in 2005–2006 [2]. The most common pneumococcal serotypes detected in children younger than 5 years of age in Hanoi between 1997 and 1999 were serotypes 23F, 19F, 6B, and 14 (ordered according to frequency) [3]. In a prospective study conducted in 2008–2009, similar serotype distributions have been observed in Vietnamese patients from all age groups, in whom the major pneumococcal serotypes were 19F, 23F, 14, 11, and 6B [4]. In Vietnam, high and increasing rates of antibiotic resistance among pneumococcal isolates have been observed, which may potentially be explained by the frequent and inappropriate use of antibiotics in that country and by the spread of multi-resistant strains [3-7].

Although routine vaccination of infants and toddlers with effective pneumococcal conjugate vaccines has been shown to be the best strategy to prevent invasive diseases caused by *Streptococcus pneumoniae* in children younger than 5 years of age, there is no pneumococcal conjugate vaccine currently available in Vietnam [8-10]. A 10-valent pneumococcal non-typeable *Haemophilus influenzae* (NTHi) protein D conjugate vaccine (*Synflorix*™, GlaxoSmithKline, Rixensart, Belgium; hereafter referred as PHiD-CV), which offers protection against pneumococcal serotypes 1, 4, 5, 6B, 7F, 9V, 14, 18C, 19F, and 23F, has been licensed in more than 110 countries across the world since 2008. Previous clinical trials conducted in various countries have shown that PHiD-CV co-administered with routinely used childhood vaccines in infants or young children was immunogenic and had a clinically acceptable safety profile [11-23].

In Vietnam, the safety and reactogenicity profile of a new vaccine needs to be evaluated in the local target population before the vaccine can be registered in the country. Therefore, the present study was designed to evaluate the safety and reactogenicity of PHiD-CV when co-administered with the licensed diphtheria, tetanus, acellular pertussis, hepatitis B virus, inactivated poliovirus, and *H. influenzae* type b vaccine (*Infanrix hexa*™, GlaxoSmithKline, Rixensart, Belgium; hereafter referred as DTPa-HBV-IPV/Hib) in a 3-dose primary vaccination course in Vietnamese infants.

## Methods

### Study objectives

The primary objective of this study was to evaluate the safety and reactogenicity of 3-dose primary vaccination with PHiD-CV co-administered with DTPa-HBV-IPV/Hib in terms of grade 3 solicited (local or general) adverse events (AEs) reported within 4 days post-vaccination and grade 3 unsolicited AEs reported within 31 days post-vaccination in Vietnamese infants.

The secondary objectives included the evaluation of the safety and reactogenicity in terms of local and general solicited AEs reported within 4 days after each vaccination, unsolicited AEs within 31 days after each vaccination, and serious adverse events (SAEs) throughout the study.

### Study vaccines

PHiD-CV (*Synflorix*™, GlaxoSmithKline, Rixensart, Belgium) contained 1 μg of each capsular polysaccharide of pneumococcal serotypes 1, 5, 6B, 7F, 9V, 14, and 23F and 3 μg of serotype 4 capsular polysaccharide conjugated individually to NTHi protein D; 3 μg of serotype 18C capsular polysaccharide conjugated to tetanus toxoid; and 3 μg of serotype 19F capsular polysaccharide conjugated to diphtheria toxoid. PHiD-CV was administered intramuscularly in the anterolateral region of the right thigh of the infants in the PHiD-CV group.

The DTPa-HBV-IPV/Hib vaccine (*Infanrix hexa*™, GlaxoSmithKline, Rixensart, Belgium) contained ≥30 IU of diphtheria toxoid, ≥40 IU of tetanus toxoid, 25 μg of pertussis toxoid, 25 μg of filamentous haemagglutinin, 8 μg pertactin, 10 μg of recombinant hepatitis B surface antigen, 40 D antigen units of poliovirus type 1, 8 D antigen units of poliovirus type 2, 32 D antigen units of poliovirus type 3, and 10 μg of Hib polysaccharide polyribosylribitol phosphate conjugated to 20–40 μg of tetanus toxoid. The DTPa-HBV-IPV/Hib vaccine was administered intramuscularly in the anterolateral region of the left thigh of the infants in both groups.

### Study design and participants

This phase III, open-label, randomised study was conducted between February 2011 and July 2011 in one of the largest tertiary hospitals of Ho Chi Minh City (the Children Hospital No. 2 [Nhi Dong 2]) in cooperation with the Clinical Research Unit of the Pasteur Institute of Ho Chi Minh City.

Study participants were recruited from the hospital vaccination clinic, where parents bring their infants for routine childhood immunisations. Eligible participants were healthy infants aged between and including 6–12 weeks (42–90 days) at the time of the first vaccination. Infants were excluded from participation if they had received previous vaccination against diphtheria, tetanus, pertussis, Hib and/or *S. pneumoniae*. Written and signed informed consent was obtained before enrolment from the parents or legally acceptable representatives (LARs) of the infants. After the parents or LARs granted informed consent, a study physician assessed the eligibility of the infants by obtaining information on their medical history and performing physical examinations. A transportation allowance was provided to participants for the fourth visit, and no other financial compensation was provided. The study was conducted in accordance with the Good Clinical Practice guidelines and the Declaration of Helsinki, and the protocol and associated documents were reviewed and approved by national and local ethics committees (Institutional Review Boards of Nhi Dong 2 Hospital and Pasteur Institute, Service of Health and People’s Committee, Ho Chi Minh City, and Independent Ethics Committee of the Vietnamese Ministry of Health). This study has been registered at http://www.clinicaltrials.gov NCT01153841. A summary of the protocol is available at http://www.gsk-clinicalstudyregister.com (GlaxoSmithKline study IDs 113151).

### Randomisation

Study participants were randomised (2:1 treatment allocation ratio) to receive 3-dose primary vaccination at 2, 3, and 4 months of age with either PHiD-CV co-administered with DPTa-HBV-IPV/Hib (PHiD-CV group) or DTPa-HBV-IPV/Hib administered alone (Control group). Infants in the PHiD-CV group received 2 injections at each vaccination visit while those in the Control group had only 1 injection per visit. The intervals between consecutive doses were to range from 28 to 42 days. Treatment allocation was performed at the investigator site using a central internet randomisation system. A randomisation blocking scheme (block size of 3) was used to ensure a balanced distribution of the infants in each group.

### Reactogenicity and safety assessment

Local (pain, redness, and swelling at injection site) and general (drowsiness, fever, irritability, and loss of appetite) symptoms were actively solicited and recorded during a 4-day period after each vaccination using diary cards that were completed by the infants’ parents or LARs. Unsolicited AEs were recorded during a period of 31 days after each vaccination. The intensity of each solicited symptom was graded on a scale from 1 to 3. Grade 3 symptoms were defined as follows: pain at the injection site if the limb was spontaneously painful or if the infant cried when it was moved, redness and swelling at the injection site if the diameter was >30 mm, fever if axillary temperature was >39.5°C, loss of appetite if the infant did not eat at all, and irritability if the infant cried and could not be comforted or if it prevented normal activity. All other AEs were considered of grade 3 intensity if they prevented normal activity. The prevalence of concomitant antipyretic (prophylactic or therapeutic) medication during the 4-day post-vaccination period was also recorded. SAEs were recorded throughout the entire study. A SAE was defined as any untoward medical occurrence that resulted in death, was life-threatening, required hospitalisation or prolongation of existing hospitalisation, or resulted in disability or incapacity. All solicited local reactions were considered causally related to vaccination as defined in the protocol. Using their clinical judgment, the investigators assessed the causality of all other AEs.

### Statistical analyses

The safety and reactogenicity analyses were performed on the total vaccinated cohort (TVC), which included all infants with at least one vaccine dose administration. In this study, 300 infants were planned to be enrolled (200 in the PHiD-CV group and 100 in the Control group). Incidences of solicited local and general symptoms during the 4-day post-vaccination period and unsolicited AEs during the 31-day post-vaccination period were calculated with exact 95% confidence interval (CI) after each vaccine dose and overall, according to the type of symptom, intensity, and relationship to vaccination. The overall incidence of AEs was calculated as the proportion of vaccine doses followed by at least one type of symptom. The prevalence of concomitant antipyretic medication during the 4-day post-vaccination period was also computed with exact 95% CI. SAEs and withdrawals due to SAEs were described in detail. This study was a descriptive study and although non-overlapping 95% CIs might indicate potential differences between study groups, these comparisons should be interpreted with caution considering that there were no adjustments for multiple comparisons of the various endpoints. The statistical analyses were performed using the SDD (i.e. Statistical Analysis System [SAS] Drug and Development) web portal version 3.5 and SAS version 9.2.

## Results

### Study participants

**Figure 1:**
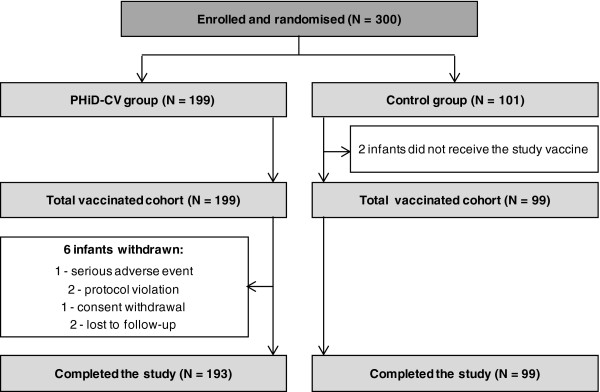
**Trial profile.** Footnote: PHiD-CV group= infants vaccinated with PHiD-CV co-administered with DTPa-HBV-IPV/Hib at 2, 3, and 4 months of age. Control group= infants vaccinated with DTPa-HBV-IPV/Hib at 2, 3, and 4 months of age. *N*= number of infants.

Between February 2011 and April 2011, 300 infants were enrolled in this study; 199 in the PHiD-CV group and 101 in the Control group (Figure 1). Of these, 199 infants in the PHiD-CV group and 99 infants in the Control group received at least one vaccine dose and were included in the TVC. The demographic characteristics of the infants included in the TVC were comparable in the 2 groups at the time of the first vaccination, although the proportion of boys seemed lower in the PHiD-CV group than in the Control group (53.3% versus 62.6%) (Table 1). All the infants were younger than 90 days of age at the time of the first dose administration. In the PHiD-CV group, the pre-specified interval between doses was exceeded by 2 infants between the first and the second dose (maximum 49 days) and by one infant between the second and the third dose (45 days). These 3 infants were included in the TVC.

**Table 1:** Demographic characteristics of participants (total vaccinated cohort)

**Characteristic**		**PHiD-CV group N=199**	**Control group N=99**
Age (weeks)	Mean ± SD	8.8 ± 1.24	8.7 ± 1.11
	Range	6–12	6–12
Gender	Female n (%)	93 (46.7)	37 (37.4)
	Male n (%)	106 (53.3)	62 (62.6)
Race	Asian – South East Asian heritage n (%)	199 (100)	99 (100)

PHiD-CV group= infants vaccinated with PHiD-CV co-administered with DTPa-HBV-IPV/Hib at 2, 3, and 4 months of age; Control group= infants vaccinated with DTPa-HBV-IPV/Hib at 2, 3, and 4 months of age; N= number of infants; n= (%) number (percentage) of infants with the specified characteristic; SD= standard deviation; Range= Minimum-Maximum values.

### Primary objective

Within 31 days post-vaccination, 8.2% of overall doses in the PHiD-CV group and 3.0% of overall doses in the Control group were followed by at least one type of grade 3 AE (solicited and/or unsolicited, local and/or general) (Table 2).

**Table 2:** Incidence of overall per dose grade 3 solicited symptoms reported within 4 days post-vaccination and of grade 3 unsolicited symptoms reported within 31 days post-vaccination (total vaccinated cohort)

	PHiD-CV group N=583/585*		**Control group N=297**	
**Grade 3 Symptom**	**n**	**% [95% CI]**	**n**	**% [95% CI]**
**Any symptom**	**48**	**8.2 [6.1, 10.8]**	**9**	**3.0 [1.4, 5.7]**
**Local symptom**	**43**	**7.4 [5.4, 9.8]**	**6**	**2.0 [0.7, 4.3]**
Pain	38	6.5 [4.7, 8.8]	3	1.0 [0.2, 2.9]
Redness	5	0.9 [0.3, 2.0]	1	0.3 [0.0, 1.9]
Swelling	7	1.2 [0.5, 2.5]	2	0.7 [0.1, 2.4]
**General symptom**	**13**	**2.2 [1.2, 3.8]**	**3**	**1.0 [0.2, 2.9]**
Drowsiness	3	0.5 [0.1, 1.5]	0	0.0 [0.0, 1.2]
Fever	0	0.0 [0.0, 0.6]	1	0.3 [0.0, 1.9]
Irritability	11	1.9 [0.9, 3.4]	1	0.3 [0.0, 1.9]
Loss of appetite	0	0.0 [0.0, 0.6]	0	0.0 [0.0, 1.2]
**Unsolicited symptom**	**2**	**0.3 [0.0, 1.2]**	**1**	**0.3 [0.0, 1.9]**

PHiD-CV group= infants vaccinated with PHiD-CV co-administered with DTPa-HBV-IPV/Hib at 2, 3, and 4 months of age; Control group= infants vaccinated with DTPa-HBV-IPV/Hib at 2, 3, and 4 months of age; N= number of doses; *= number of administered doses for unsolicited symptom; n= number of reported symptoms; %= percentage of doses followed by the specified symptom with grade 3 intensity; 95% CI= 95% confidence interval.

Within 4 days post-vaccination, pain at the injection site was the most frequently reported grade 3 solicited local symptom, which was reported following 6.5% and 1.0% of overall doses in the PHiD-CV and Control groups, respectively. In the PHiD-CV group, the most common grade 3 solicited general symptom was irritability, which was reported following 1.9% of overall doses and was considered causally related to vaccination following 1.7% of overall doses. In the Control group, irritability and fever were the only grade 3 solicited general symptoms and each symptom was reported following one dose (0.3% of overall doses). Grade 3 fever that was considered causally related to vaccination was reported following one dose (0.3% of overall doses) in the Control group.

In both groups, grade 3 unsolicited symptoms were uncommon; they were reported following 2/585 doses (constipation and fungal infection; 0.3% of overall doses) in the PHiD-CV group and following 1/297 doses (fungal infection; 0.3% of overall doses) in the Control group. None of the grade 3 unsolicited symptoms were considered causally related to vaccination.

### Secondary objectives

#### Solicited symptoms

**Figure 2:**
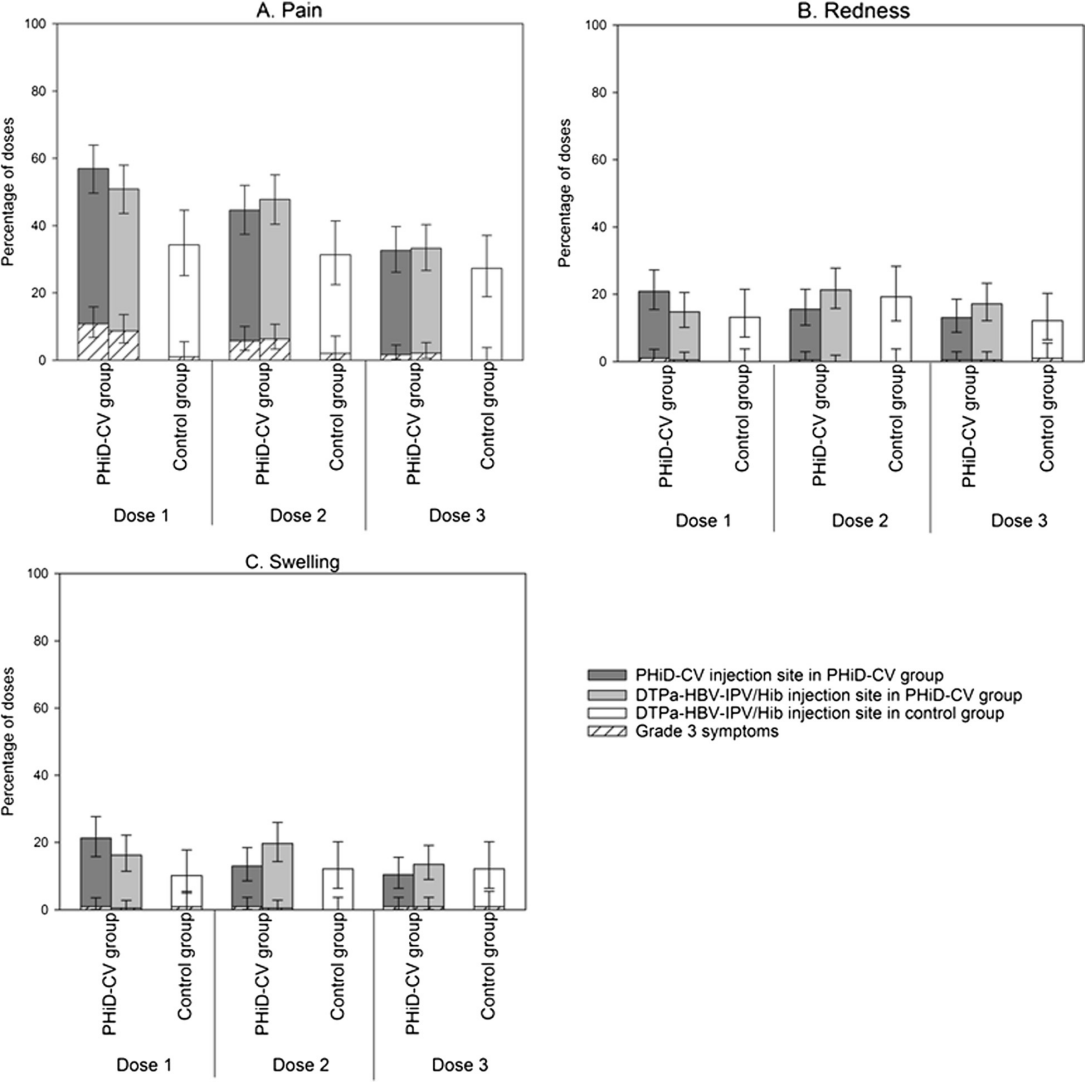
**Percentages of doses followed by pain (A), redness (B), and swelling (C) at the specified injection site of any intensity and grade 3 intensity reported during the 4-day post-vaccination period (total vaccinated cohort).** Footnote: PHiD-CV group= infants vaccinated with PHiD-CV co-administered with DTPa-HBV-IPV/Hib at 2, 3, and 4 months of age. Control group= infants vaccinated with DTPa-HBV-IPV/Hib at 2, 3, and 4 months of age. Grade 3 symptoms: pain if the infant was crying when the limb was moved or if the limb was spontaneously painful and redness or swelling if the diameter was >30 mm. Error bars indicate 95% confidence intervals.

**Figure 3:**
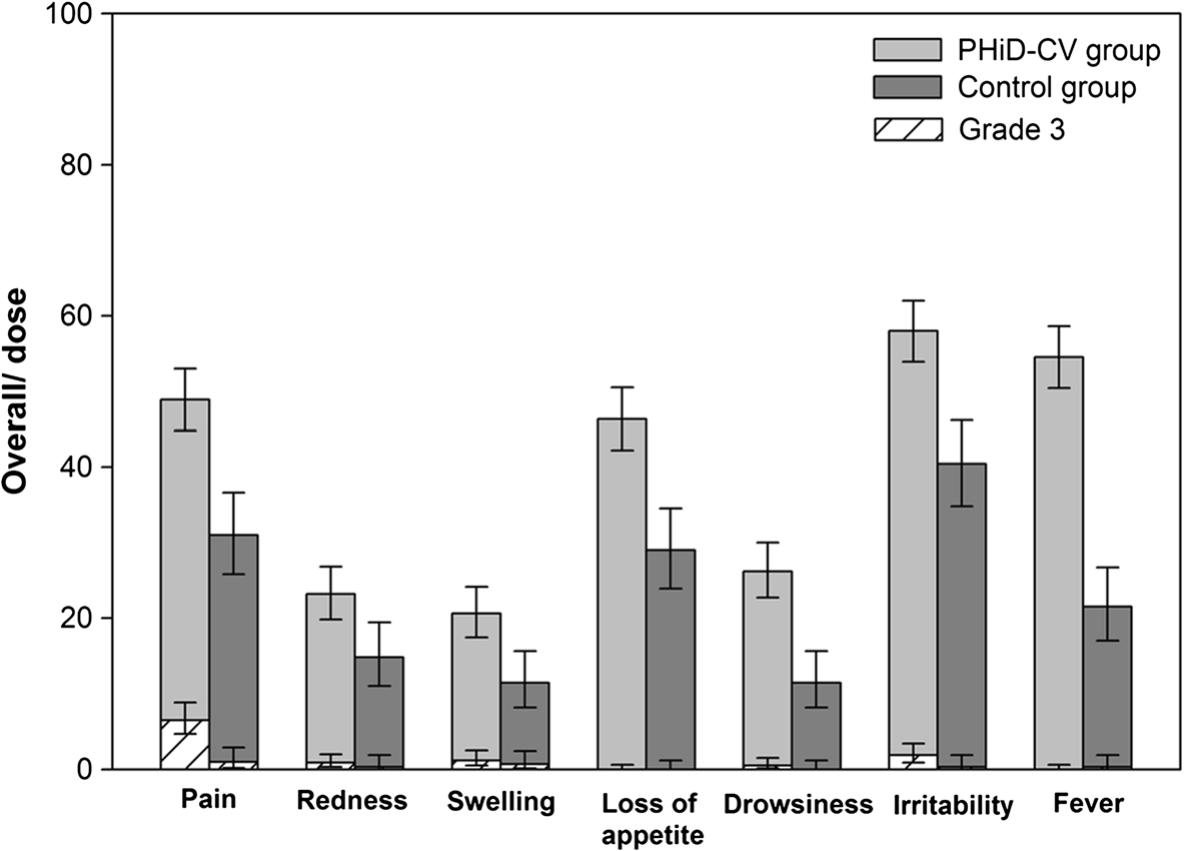
**Incidence of overall per dose solicited local (pain, redness, and swelling) and general (drowsiness, fever, irritability, and loss of appetite) symptoms of any intensity and grade 3 intensity reported during the 4-day post-vaccination period (total vaccinated cohort).** Footnote: PHiD-CV group= infants vaccinated with PHiD-CV co-administered with DTPa-HBV-IPV/Hib at 2, 3, and 4 months of age. Control group= infants vaccinated with DTPa-HBV-IPV/Hib at 2, 3, and 4 months of age. Grade 3 symptoms: pain if the infant was crying when the limb was moved or if the limb was spontaneously painful, redness or swelling if the diameter was >30 mm, drowsiness if it prevented normal activity, fever if axillary temperature was >39.5°C, irritability if the infant was crying and could not be comforted or if it prevented normal activity, and loss of appetite if the infant did not eat at all. Error bars indicate 95% confidence intervals.

During the 4-day post-vaccination period, the most frequently reported solicited local symptom following each vaccine dose was pain at the injection site (Figure 2). Overall, pain was reported following 48.9% and 31.0% of doses, redness following 23.2% and 14.8% of doses, and swelling following 20.6% and 11.4% of doses in the PHiD-CV and Control groups, respectively (Figure 3). In the PHiD-CV group, the incidence of each solicited local symptom was comparable at both injection sites (PHiD-CV and DTPa-HBV-IPV/Hib) after each vaccine dose (Figure 2) and the overall per dose frequencies of symptoms at each injection site were within similar ranges (data not shown).

**Figure 4:**
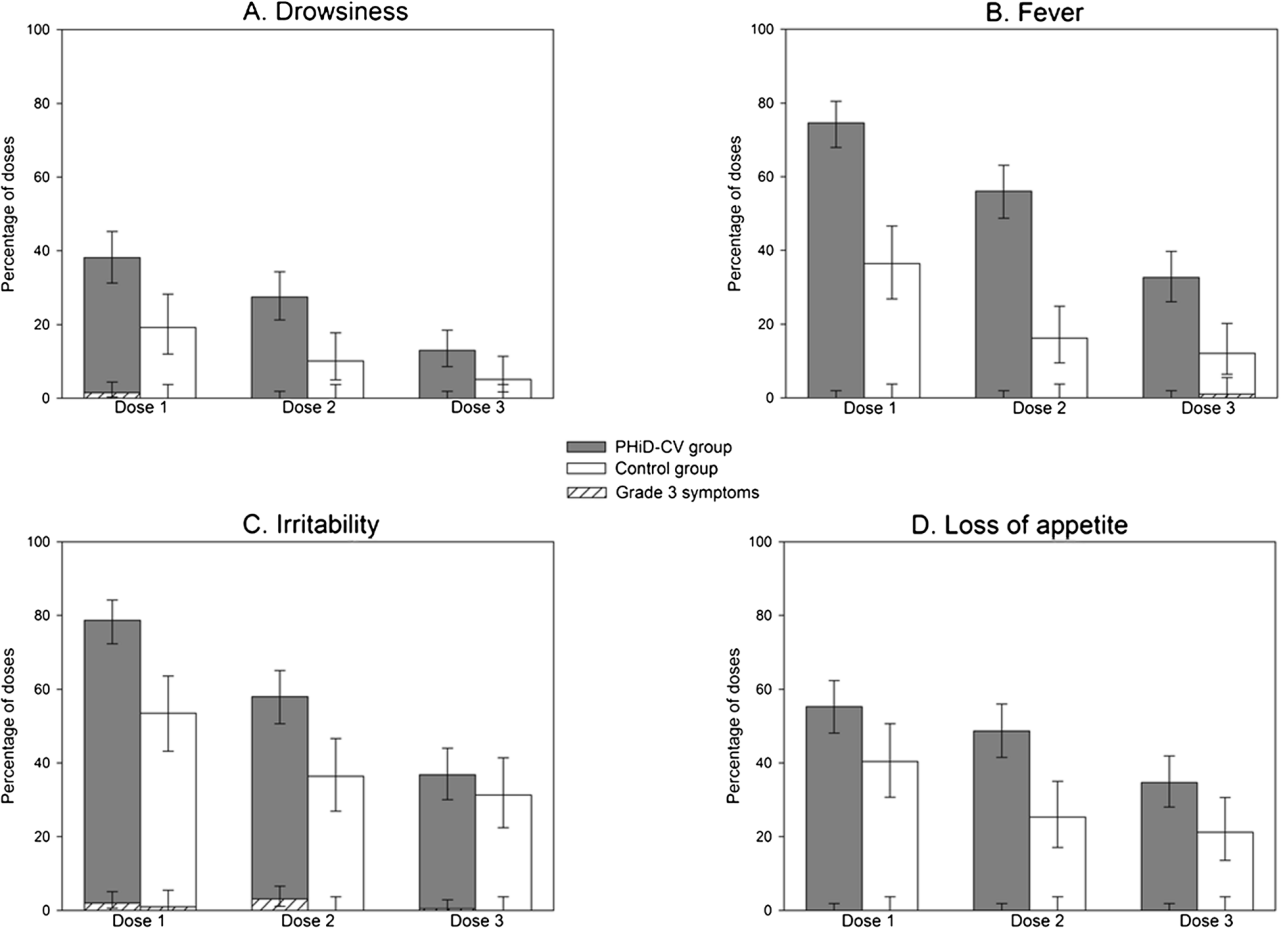
**Percentages of doses followed by drowsiness (A), fever (B), irritability (C), and loss of appetite (D) of any intensity and grade 3 intensity reported during the 4-day post-vaccination period (total vaccinated cohort).** Footnote: PHiD-CV group= infants vaccinated with PHiD-CV co-administered with DTPa-HBV-IPV/Hib at 2, 3, and 4 months of age. Control group= infants vaccinated with DTPa-HBV-IPV/Hib at 2, 3, and 4 months of age. Grade 3 symptoms: drowsiness if it prevented normal activity, fever if axillary temperature was >39.5°C, irritability if the infant was crying and could not be comforted or if it prevented normal activity, and loss of appetite if the infant did not eat at all. Error bars indicate 95% confidence intervals.

In both groups, the most frequently reported solicited general symptom following each vaccine dose was irritability (Figure 4). The overall per dose incidence of solicited general symptoms seemed to be higher in the PHiD-CV group compared with the Control group (irritability: 58.0% versus 40.4%; fever: 54.5% versus 21.5%; loss of appetite: 46.3% versus 29.0%; and drowsiness: 26.2% versus 11.4%) (Figure 3).

Antipyretic medication use within 4 days post-vaccination was infrequent in both groups and was reported following 3.2% (95% CI: 2.0–5.0) of overall doses in the PHiD-CV group and following 1.0% (95% CI: 0.2–2.9) of overall doses in the Control group (data not shown).

#### Unsolicited symptoms

Within 31 days post-vaccination, unsolicited AEs were reported following 12.3% (95% CI: 9.8–15.2) and 14.8% (95% CI: 11.0–19.4) of doses in the PHiD-CV and Control groups, respectively (data not shown). The most frequently reported unsolicited symptoms were upper respiratory tract infection (following 3.8% [95% CI: 2.4–5.6] and 3.0% [95% CI: 1.4–5.7] of doses in the PHiD-CV and Control groups, respectively) and bronchiolitis (following 1.9% [95% CI: 0.9–3.3] and 3.4% [95% CI: 1.6–6.1] of doses in the PHiD-CV and Control groups, respectively).

Unsolicited symptoms considered causally related to vaccination were reported following one dose in the PHiD-CV group (rash) and following one dose in the Control group (vomiting). Unsolicited symptoms with medically attended visit were reported following 9.4% (95% CI: 7.2–12.1) of doses in the PHiD-CV group and 8.4% (95% CI: 5.5–12.2) of doses in the Control group.

#### Serious adverse events

Throughout the entire study period, 13 SAEs were reported in 9 infants in the PHiD-CV group: convulsion, diarrhoea, bronchiolitis (n=2 for each SAE), autoimmune thrombocytopenia, upper respiratory tract infection, gastro-oesophageal reflux disease, hydronephrosis, fungal infection, Kawasaki’s disease, and coagulopathy (n=1 for each SAE). In the Control group, 11 SAEs were reported in 6 infants: bronchiolitis (n=3), diarrhoea, fungal infection (n=2 for each SAE), urinary tract infection, gastro-oesophageal reflux disease, oral candidiasis, and viral infection (n=1 for each SAE). None of these SAEs were fatal or considered to be causally related to vaccination.

## Discussion

In Vietnam, the reactogenicity and safety of a new vaccine need to be evaluated in the local target population before the vaccine can be registered in the country. In the present study, the reactogenicity and safety profile of PHiD-CV co-administered with DTPa-HBV-IPV/Hib in Vietnamese infants seemed in line with that observed in previous studies, in which PHiD-CV was co-administered with routine childhood vaccines [13,21-23].

Within the 31-day post-vaccination period, 8.2% of overall doses were followed by at least one grade 3 symptom (solicited and/or unsolicited, local and/or general) in the infants who received PHiD-CV co-administered with DTPa-HBV-IPV/Hib, while this was 3.0% of overall doses in the infants who received DTPa-HBV-IPV/Hib alone. This difference was mainly due to the higher proportion of doses followed by grade 3 pain during the 4-day post-vaccination period in the infants who received both vaccines co-administered compared with those who received DTPa-HBV-IPV/Hib alone (6.5% [95% CI: 4.7, 8.8] versus 1.0% [95% CI: 0.2, 2.9] of overall doses). This difference in incidence of pain was not unexpected and could be explained by the fact that the infants of the PHiD-CV group had 2 injections at each vaccination visit while those in the Control group had only 1 injection per visit. The most frequently reported grade 3 solicited general symptom in the infants who received PHiD-CV co-administered with DTPa-HBV-IPV/Hib was irritability, which was reported following 1.9% (95% CI: 0.9, 3.4) of doses. The use of antipyretic medication was low in both groups and grade 3 fever was only reported in one infant vaccinated with DTPa-HBV-IPV/Hib alone.

The incidence and nature of the grade 3 solicited symptoms reported in this study were in line with those reported in a previous study assessing the co-administration of PHiD-CV and DTPa-HBV-IPV/Hib in Taiwanese infant. In that study, the most frequently reported grade 3 solicited local and general symptoms were pain (following 4.2% of doses [95% CI: 2.8, 6.0]) and irritability (following 6.3% of doses [95% CI: 4.6, 8.4]) [21]. In another study, in which PHiD-CV was co-administered with a monovalent vaccine against Hib in Korean infants, grade 3 solicited symptoms were reported following no more than 2.6% of doses, which seems slightly lower than the reporting rates observed in the present study [22]. When compared to co-administration of PHiD-CV and DTPa-based or Hib vaccines, the incidence of grade 3 symptoms seemed higher when PHiD-CV was co-administered with whole cell pertussis-based combination vaccines, as observed in 2 Asian studies, in which the incidence of grade 3 pain reached 9.4% (95% CI: 6.4, 13.3) and 27.7% (95% CI: 24.4, 31.1) of doses and grade 3 irritability, 2.9% (95% CI: 1.9, 4.2) and 8.3% (95% CI: 6.4, 10.6) of doses in infants in the Philippines and India, respectively [13,23]. This observation was expected since whole cell pertussis-based vaccines are known to induce more adverse events than DTPa-based vaccines [24].

In the present study, pain was the most frequently reported solicited local symptom with any intensity, which was reported following 48.9% (95% CI: 44.8, 53.0) of PHiD-CV doses co-administered with DTPa-HBV-IPV/Hib. In contrast, redness was the most common solicited local symptom in previous Asian studies conducted in Taiwan and Korea, in which PHiD-CV was also co-administered with DTPa-based vaccines [21,22]. In the previous studies conducted in India and the Philippines, pain was also the most common solicited local symptom following co-administration of PHiD-CV and whole cell pertussis-based vaccines (following 71.0% of doses [95% CI: 67.6, 74.4] and 67.2% of doses [95% CI: 64.0, 70.3]) [13,23]. The incidence of solicited local symptoms was comparable at both injections sites in the infants who received PHiD-CV co-administered with DTPa-HBV-IPV/Hib, but solicited local and general symptoms seemed more frequently reported in infants who received PHiD-CV in addition to DTPa-HBV-IPV/Hib than in those who received DTPa-HBV-IPV/Hib alone.

The percentage of doses followed by at least one unsolicited adverse event within the 31-day post-vaccination period was comparable in both groups and the incidences of grade 3 unsolicited symptoms were low. Unsolicited symptoms causally related to vaccination were only reported in one child in each group. Throughout the study, 15 infants (9/199 infants in the PHiD-CV group and 6/99 infants in the Control group) reported at least one SAE. None of these SAEs were fatal or considered by the investigator to be causally related to vaccination.

This study had a very high compliance rate since 97.0% of the infants who received PHiD-CV co-administered with DTPa-HBV-IPV/Hib and all the infants vaccinated with DTPa-HBV-IPV/Hib alone received the 3 vaccine doses. However, potential limitation of the study included its non-blinded (open) design, due to the different number of injections in both groups. The open design might have induced a bias in the reactogenicity and safety profile towards increased reporting of AEs in infants in the PHiD-CV group since their parents or LARs and the investigators were aware that they had received a new vaccine in addition to the antigens received by the infants in the Control group. Another limitation of this study was the fact that there were no confirmatory analyses with regards to the reactogenicity and safety objectives. Due to above mentioned limitations, the study results should be interpreted with caution.

## Conclusions

This study showed that 3-dose primary vaccination with PHiD-CV co-administered with DTPa-HBV-IPV/Hib at 2, 3, and 4 months of age had a clinically acceptable reactogenicity and safety profile in Vietnamese infants, which was in line with that observed in other South-East Asian populations [13,22]. These results lend support for the introduction of PHiD-CV in routine paediatric vaccination programmes in Vietnam, a country where no pneumococcal conjugate vaccines are currently available and where young children could have substantial benefit from immunisation against pneumococcal disease.

Synflorix and Infanrix hexa are registered trademarks of the GlaxoSmithKline group of companies.

## Abbreviations

AE: Adverse event;CI: Confidence interval;DTPa-HBV-IPV/Hib: Diphtheria, tetanus, acellular pertussis, hepatitis B virus, inactivated poliovirus, and *Haemophilus influenzae* type b vaccine;Hib: *Haemophilus influenzae* type b;LAR: Legally acceptable representative;NTHi: Non-typeable *Haemophilus influenzae*;PHiD-CV: 10-valent pneumococcal non-typeable *Haemophilus influenzae* protein D conjugate vaccine;SAE: Serious adverse event;TVC: Total vaccinated cohort

## Competing interests

TNH was the Principal Investigator for this study conducted in Vietnam. GlaxoSmithKline Biologicals SA was the funding source and was involved in all stages of the study conduct and analysis. NTT, HMT and HLV declare no conflict of interest. PLTB received funding for the study from GlaxoSmithKline Biologicals SA. FS is employed by GlaxoSmithKline group of companies. TWY, DB, and AH are employed by GlaxoSmithKline group of companies and have stock/stock options ownership.

## Authors’ contributions

TNH, HMT, NTT, HLV, AH, and DB were involved in the planning and design of the reported study; TNH, TWY, AH, and DB, in the review and finalisation of the reported study; AH, in the overall follow-up of the study; TNH, in the choice and recruitment of investigators and in the coordination of the study; PLTB, HMT, and HLV, in the provision of subjects; TNH, HMT, HLV, PLTB, NTT, and TWY, in the collection of the data; TNH, HMT, NTT, TWY, AH, and DB, in the interpretation of the results; HMT, in supervising the analysis; HLV and PLTB, in the acquisition of funding; TNH and PLTB, in the supervision of the study group; and FAS, in statistical analysis and data management. All authors reviewed the different drafts of the manuscript and approved the final version.

## Pre-publication history

The pre-publication history for this paper can be accessed here:

http://www.biomedcentral.com/1471-2334/13/95/prepub
